# Inoculation of Pan02 cells produces tumor nodules in mouse pancreas: Characterization of a novel orthotopic pancreatic ductal adenocarcinoma tumor model for interventional studies

**DOI:** 10.1371/journal.pone.0300723

**Published:** 2024-03-28

**Authors:** James I. Griffin, Xinyue Chen, Luqi Duan, Qingxin Mu, Rodney J. Y. Ho

**Affiliations:** 1 Departments of Pharmaceutics, University of Washington, Seattle, Washington, United States of America; 2 Departments of Bioengineering, University of Washington, Seattle, Washington, United States of America; Affiliated Hospital of Nanjing University of Chinese Medicine: Jiangsu Province Academy of Traditional Chinese Medicine, CHINA

## Abstract

Preclinical models of cancer are vital for assessing and predicting efficacies and toxicities of novel treatments prior to testing in human subjects. Current pancreatic tumor models exhibit variable growth rates, unpredictable tumor size after implantation in non-native tissues, or require surgical implantation. Surgical implantation in the pancreas may produce not only unpredictable tumor uptake but could also elicit additional inflammatory responses. In searching for a pancreatic carcinoma cell that can be introduced into a mouse via simple injection, we found that Pan02, a murine ductal pancreatic adenocarcinoma derived from a pancreatic lesion of a C57BL/6 mouse, inoculated peritoneally can consistently produce pancreatic tumors. This intraperitoneal, but not intravenous, introduction of Pan02 cells leads to the attachment and growth of Pan02 in the pancreas before spreading to other tissues. Time-course tissue analysis indicates that the Pan02 cells first find, infiltrate, and grow within the pancreas, producing a pancreatic tumor model. This model appears to mimic pancreatic cancer development in humans and is the first reported use of Pan02 cells to produce orthotopic pancreatic and metastatic neoplasms in a mouse model without the need for tumor implantation within matrices or survival surgeries. This orthotopic pancreatic tumor model, with consistent tumor uptake, synchronized tumor development and survival, and predictable outcomes may enable and accelerate the preclinical evaluation of treatment candidates for pancreatic cancer.

## Introduction

While modern small molecule and biologic therapies extend the life of patients with many cancers, these advances have had a limited impact on pancreatic cancer. Fewer than 50% of patients with pancreatic cancer live beyond 1 year; ~5% for 5 years [1]. Even with its relatively low incidence, the annual death rate for pancreatic cancer is one of the highest for all cancers as its annual incidence is roughly equivalent to its death rate [2]. In contrast, advances in breast cancer therapy have led to 300,000 reported cases per year with only ~45,000 reported deaths. More than 85% of pancreatic cancers are attributed to ductal adenocarcinoma. The challenges to pancreatic treatment success are due, in part, to (1) the fact that high density tumor mass resides within pancreatic tissue (typically encased with the overgrown extracellular matrix proteins, a condition referred to as desmoplasia) [3], (2) the difficulty of macromolecules—such as monoclonal antibodies delivered in the blood—reaching the interior of pancreatic tumor nodules with limited blood perfusion, (3) limited tissue distribution of drugs through blood vessels, and (4) limited information on druggable targets for pancreatic cancer. Numerous antibody therapeutics—targeted to specific and other cancer cell surface markers—are adapted for pancreatic cancer treatment, including trastuzumab targeted to HER2 (human epidermal growth factor receptor 2, which is effective for breast cancer patients expressing the HER2 marker). (Trastuzumab, for example was first approved for breast cancer and adapted later to evaluate as a candidate for pancreatic cancer treatment.) However, due to evolving pancreatic tumor markers, trastuzumab alone or in combination with gemcitabine has not yielded a significant impact [4, 5]. While pancreatic tumor cells may express some of these druggable molecular targets, low-to-moderate levels of target-protein expression mean that the drugs that work for other types of cancer may be insufficient to improve pancreatic cancer treatment outcomes.

While pancreatic tumor markers are being developed and verified, current pancreatic cancer treatments are relegated to broadly cytotoxic yet highly potent small molecules, in a combination that inhibits cell growth and the replication functions of rapidly growing cancer cells. Due to the non-specific drug distribution throughout the body, patients experience significant, intolerable, and off-target untoward side-effects [6–8]. Therefore, there is an urgent need to find safe, effective, and potent treatment products for pancreatic cancer. To evaluate drug candidates, each promising product must undergo rigorous preclinical evaluation that progresses to verify safety and efficacy in appropriate animal models that mimic pancreatic tumors. Unfortunately, there are limited preclinical animal tumor models reflective of disease development within the pancreas and how it spreads metastatically to other tissues. In addition, a pancreatic tumor model that can be consistently synchronized for predictable tumor growth in physiologic conditions would be needed to discern treatment effects in a reproducible manner. Having such a pancreatic cancer model will enable the accelerated selection of the most promising drug and biologic candidates to specifically develop treatments effective for pancreatic cancer. Such a model is currently lacking or otherwise underdeveloped, thus partly impeding the disparate progress made in pancreatic cancer treatment.

While genetic knockout pancreatic tumor models are available, they may not provide consistent, predictable tumor growth and are limited to selecting genes altered in pancreatic cells. Human pancreatic tumor cells implanted in nude mice provide a xenograft, which is typically presented as a subcutaneous (not a pancreatic) tumor. Surgical implantation is needed to produce a tumor mass in the pancreas; however, this approach may not be easily scaled for interventional studies. Surgical procedures may also pose inflammatory concerns. Similar technical and physiological considerations also apply for the use of patient-derived pancreatic cells implanted in immune-deficient mice or PDX models. In evaluating a drug candidate’s ability to arrest and kill pancreatic tumors, a contribution to the host immune system may need consideration [9]. The lack of an intact immune system removes this element, which may add uncertainty in extrapolating therapeutic potential effectiveness in suppressing pancreatic cancer. A preclinical pancreatic tumor model reflective of tumor pathophysiology and disease progression in mice with competent immune systems initiating from pancreatic tissue may address the above mentioned challenges in evaluating therapeutic candidates for pancreatic cancer. Having a simplified and reproducible pancreatic tumor in physiological contexts within the pancreas or an orthotopic tumor model may enable preclinical evaluation of drug and immunotherapeutic agents with the efficiency needed to advance treatments intended for pancreatic cancer.

Therefore, we have evaluated whether pancreatic ductal adenocarcinoma (Pan02) cells, when inoculated in syngeneic C57BL/6 mice, lead to the formation of pancreatic tumor nodules in a consistent manner. The Pan02 (also known as Panc02 immortalized) cells are originally derived from cancerous pancreatic ductal tissue in C57BL/6 mice induced with 3-methylcholartene carcinogens [10]. Unexpectedly, we found that intraperitoneal, and not intravenous, inoculation of Pan02 cells resulted in consistent Pan02 tumor uptake and growth to tumor nodules starting in the pancreas before spreading to other tissues. Due to the consistent and nearly 100% tumor take-rate of Pan02 cells, this approach may serve as a method of providing an orthotopic pancreatic tumor in mice that mimics ductal pancreatic cancer.

## Materials and methods

### Reagents and cell line

Gemcitabine (“G”; CAS: 95098-81-4) was purchased from LC Laboratories (Woburn, MA). Pan02 cells were purchased from the National Cancer Institute (NCI) Division of Cancer Treatment and Diagnosis (DCTD) Tumor Repository (NCI, Frederick, MD). Luciferase expressed Pan02 or Pan02-luc cells were a generous gift from Dr. Mien-Chie Hung of the MD Anderson Cancer Center. RPMI 1640, DMEM, F12 tissue culture medium, Hank’s Buffered Salt Solution, and fetal bovine serum (FBS), Antibiotic-Antimycotic (100X) (Anti-Anti) are from Gibco^™^ (Thermo Fisher Scientific, Waltham, MA). G418 sulfate (a neomycin analogue; Fisher Scientific, Pittsburgh, PA). D-Luciferin potassium salt (XenoLight) was purchased from PerkiElmer (Waltham, MA). Other reagents, including normal saline, and solvents were of analytical grades or higher. When analyzing the activity of luciferase and the sensitivity of Pan02-luc cells toward gemcitabine, Pan02-luc cells were cultured in the same media as Pan02 cells (that is, Gibco^™^ RPMI 1640 Medium (1X) with 10% FBS and 1% Anti-Anti).

### Verification of Pan02-luc cells’ luminescence signal in cell culture

Pan02-luc cells, described above, were seeded in a black 96-well plate (Corning Inc., Corning, NY) at 8 different densities (80,000, 40,000, 20,000, 10,000, 5,000, 2,500, 1,250 and 610 cells per well) in RPMI with 10% FBS with 1% Anti-Anti, and set to grow overnight. Wells with media only were used as negative controls. Varying D-luciferin (300, 150, 30, 5, and 0 μg/mL) concentrations were added to cell culture media. Fifteen minutes following the introduction of D-luciferin to the cells, the culture plate was placed into a Lumina II IVIS (*In Vivo* Imaging System) instrument (PerkinElmer, Waltham, MA) to measure luminescence over a 30 second period. Bioluminescence data along with digitized images were collected and integrated using instrument-associated Living Image software.

### Effect of luciferase on *in vitro* sensitivity of Pan02 cells towards gemcitabine free drug

Pan02 cells and Pan02-luc cells were seeded in a 96-well plate with 5000 cells per well and set to grow overnight. Cells were cultured in 100 μL of media containing varying concentrations of gemcitabine (range: 0-100ng/mL) for 4 days. Drug treatment effects were determined using an AlamarBlue Cell Viability Assay (Thermo Fisher Scientific, Waltham, MA). AlamarBlue was diluted 10-fold with cell media; the existing cell culture media in the plates was replaced with the 10% AlamarBlue media and allowed to incubate for 4 h. The cell culture media was then assessed for fluorescence (λ_ex_ = 570 nm, λ_em_ = 585 nm) with a PerkinElmer 1420 Multilabel Counter plate reader. Dose-response curves were fitted employing GraphPad software with an Emax model to estimate mid-point of drug concentration that reduced growth of cells by 50%, also known as EC_50_ values.

### Route of Pan02 inoculation on tumor establishment and pancreatic tissue homing in mice

All animal studies were conducted in accordance with University of Washington Institute of Animal Care and Use Committee (IACUC) approved protocols (#2372–06) as well as with federal guidelines, including power analysis to ensure that sufficient number of animals are enrolled in each group to provide statistical power to discern test vs control treatments. Four-week-old, C57BL/6 (SPF free) female mice from the Jackson Laboratory (Bar Harbor, Maine) (n = 4 per group) were intraperitoneally inoculated with 10 million Pan02-luc cells (suspended in 200uL of sterile Hank’s Buffered Salt Solution) or intravenously dosed with 5 million Pan02-luc cells (suspended in 100uL of sterile Hank’s Buffered Salt Solution, HBSS) through the tail vein. Mouse health was assessed through a combination of IVIS imaging, daily weight monitoring and body scoring. Bioluminescence of Pan02-luc in mice, under anesthesia (with isoflurane) was examined by the IVIS instrument. Mice received 150 mg/kg D-luciferin through intraperitoneal injections 12 minutes before imaging. The bioluminescence imaging parameters for living mice were set as follows: field of view, 12; excitation filter, closed; emission filter, open; exposure time, 120 sec; binning factor, 4; f/stop, 2. Total Pan02-luc bioluminescence emission from living mice was integrated using the Live Image software. Live mouse IVIS images were taken every 3 days until either 28 days following initial inoculation or before the mice reached euthanasia criteria (excessive tumor burden of more than 10% of body mass, more than a 20% loss of body weight, or other obvious indicators of suffering). Mice were sacrificed by CO_2_ under anesthesia. The organs and tissues were dissected after mice were euthanized and transferred to culture/Petri dishes, rinsed in PBS, and soaked in a 10% formalin solution for 5 minutes. Tissue images were acquired using an exposure time of 30 seconds.

### Effects of cell number on pancreatic tumor development in mice

Varying amounts (1, 5, or 10 million) of Pan02-luc cells in 200μL sterile HBSS were inoculated to C57BL/6 mice (n = 3) via an intraperitoneal route. Mouse health was assessed through a combination of weight monitoring, body scoring, and IVIS imaging as described above. Mouse imaging with IVIS took place every 3 days until either 28 days following initial inoculation or before the mice reached euthanasia criteria. Organ and tissue IVIS images were taken as described above.

#### Histopathological analysis and serum biochemistry in mice inoculated with Pan02 pancreatic ductal adenocarcinoma cells

Five million Pan02-luc cells (suspended in 200 μL HBSS) were inoculated intraperitoneally to C57BL/6 mice. They were then randomly divided into 3 (n = 4) groups. Mouse health was assessed through a combination of weight monitoring, body scoring, and IVIS imaging. Three groups of mice were sacrificed at 7 days, 14 days, and 21 days after inoculation respectively. Mouse tissues and blood were harvested. IVIS images of tissues were taken as described above. After images were taken, the tissues were placed into 10% neutral-buffered formalin (Richard-Allan Scientific, Kalamazoo, Michigan, USA) for 24 hours to fix the tissues. Tissues were then washed with 70% ethanol in water for 6 hours with changes every 2 hours. These fixed tissues were then sent to Histology Consultation Services (Everson, WA) for H&E staining and Mass trichrome staining. The formalin-fixed tissues were analyzed with NIH Elements BR 5.11.01 software on a Nikon Eclipse Ti microscope (Nikon, Tokyo, Japan). Blood samples were spun down at 1,000 G for 15 minutes at 4°C to isolate serum. Serum was submitted to Moichor (San Francisco, CA) for serum biochemistry analysis. The data were presented with reference values based on previous work from Loeb, et al. [11].

### Statistical analysis

Data were presented as mean ± standard deviation (SD). Students’ t-tests were used to analyze between two-group and ANOVA for multiple group analysis. A P-value of <0.05 was considered statistically significant. Statistical analyses were performed using GraphPad Prism (Version 9.0).

## Results

### Characterization of pancreatic ductal adenocarcinoma Pan02 cells expressing luciferase marker

**Fig 1:**
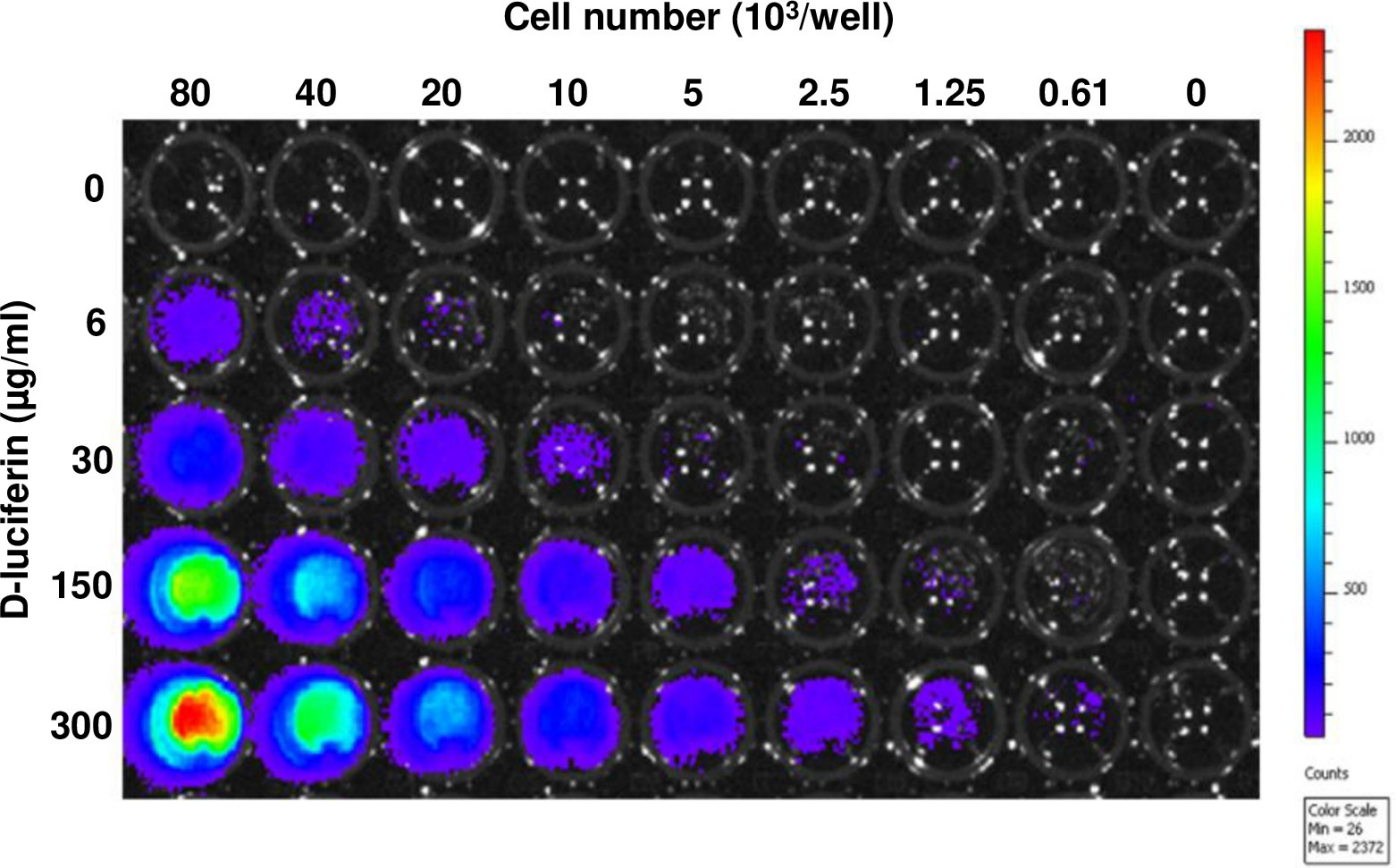
Assessment of luciferase activity in Pan02 cells. The activity of luciferase expressed in Pan02 cells was assessed in a 96-well plate. Pan02-luc cells were serially diluted and the corresponding cell numbers were labeled at the top of the graph. The concentration of D-luciferin was indicated on the left side. After adding D-luciferin, the bioluminescence intensity of each well was measured using the IVIS Spectrum System for 30 seconds, 15 minutes post-addition. The Color scale: Min = 26; Max = 2372. This 96-well plate is a representative of triplicate plates tested.

To verify the functional activity of luciferase enzyme marker expression, we first evaluated Pan02-luc cells’ ability to catalyze D-luciferin substrate into luminescence product. As shown in Fig 1, we found that luciferase enzymes in Pan02-luc cells are active, leading to luminescence increase in a cell-concentration dependent manner (Fig 1). Effects of luciferin concentration analysis indicated that 6μg/mL D-luciferin was sufficient to detect 40x10^3^ Pan02-luc cells. At 300 μg/mL luciferin, about 40 fold lower, 1,000 Pan02-luc cells were detectable. For *in vivo* mouse studies we thus used 30 mg/mL, over three orders of magnitude higher than the minimal *in vitro* D-luciferin concentrations, to enable real-time *in vivo* tumor tracking for Pan-02 growth.

### Sensitivity of Pan02 cells to pancreatic cancer drug gemcitabine

We next determined Pan02 cells’ sensitivity to the current pancreatic cancer drug, gemcitabine. To do so, we also evaluated whether having luciferase expressed in Pan02 impacted gemcitabine sensitivity. The results summarized in Table 1 indicate that both Pan02 cells and Pan02-luc cells exhibit similar sensitivity to gemcitabine. Based on a series of dose-response studies, the effective half-maximal inhibitory concentration, EC_50_, estimated for Pan02 and Pan02-luc were 9 ± 0.03 and 7 ± 1.1 ng/mL (equivalent to 34 and 26.7 nM; p>0.05), respectively. The data indicate that both Pan02 cells exhibit similar sensitivity to gemcitabine.

**Table 1:** Sensitivity of Pan02 parent and Pan02-luciferase cells to pancreatic tumor drug, gemcitabine.

Cell	Gemcitabine EC_50_	
	Mean ± SD (ng/mL)	Mean ± SD (nM)
**Pan02**	9.0 ± 0.03	34.2 ± 0.11
**Pan02-luc**	7.0 ± 1.1	26.7 ± 4.2

The gemcitabine sensitivity data, based on dose-dependent inhibition curve are fitted based on E_max_ model and expressed as half-maximum Effective Concentration or EC_50_. The replicate EC_50_ data were presented as Mean and a SD.

### Effects of Pan02 inoculation route on pancreatic tumor development in mice

**Fig 2:**
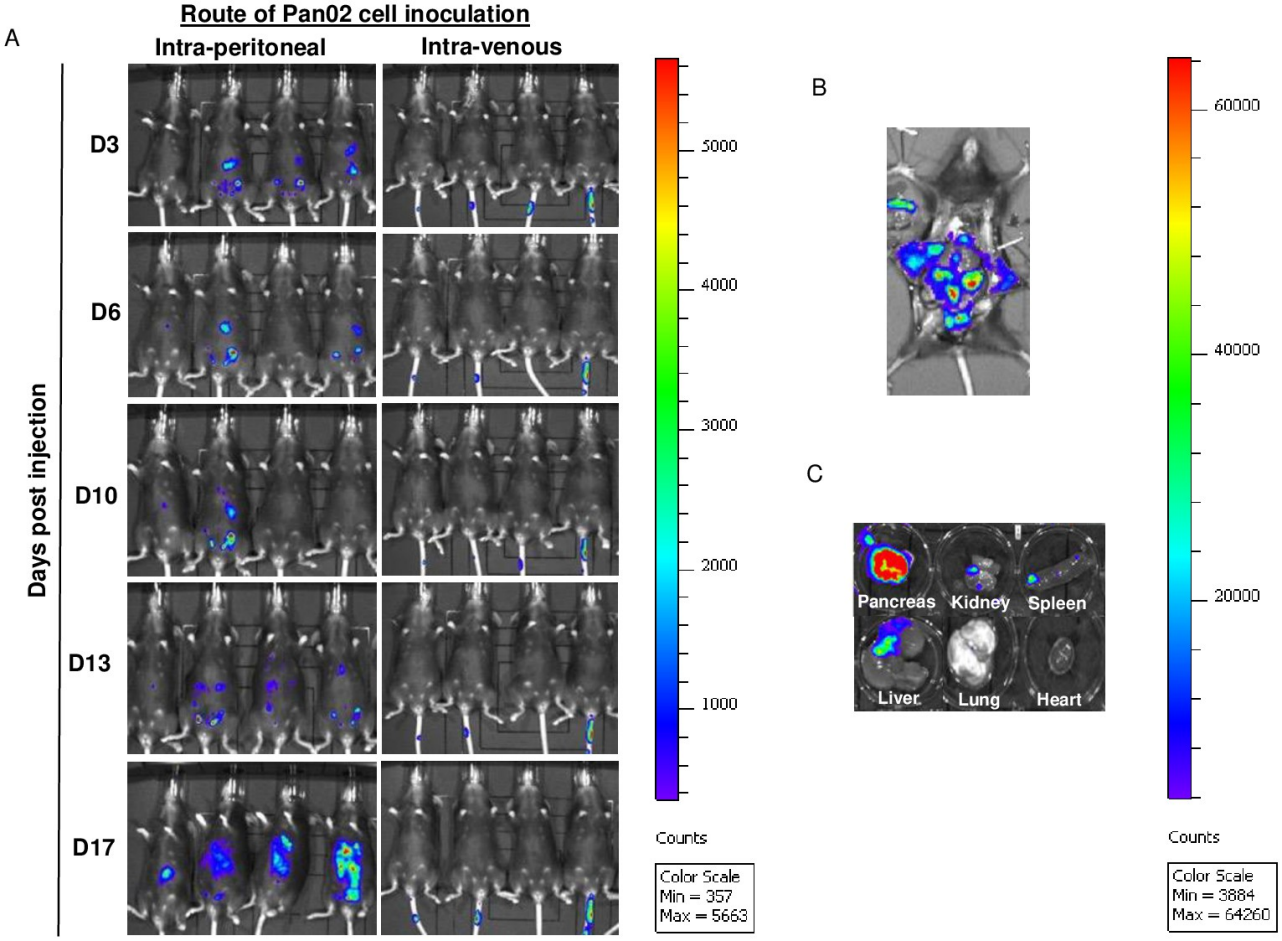
Route of Pan02 inoculation and effects on tumor establishment. C57BL/6 mice were divided into two groups, the first group (n = 4) received intraperitoneal inoculation of 10 million Pan02-luc cells, the second group (n = 4) received an intravenous inoculation of 5 million Pan02-luc cells. (A) Whole body Bioluminescence images of the two groups of mice were captured on days 3, 6, 10, 13, and 17 after inoculation using the IVIS Spectrum System. Images were taken 15 minutes after intraperitoneal injection of D-luciferin, with a 3-minute exposure. The Color scale: Min = 375; Max = 5663. (B) Bioluminescence images of laparotomy and (C) organs of mouse after intraperitoneal inoculated 10 million Pan02-luc cells 17 days from a representative mouse. The Color scale: Min 3884; Max = 64260.

To determine whether non-surgical inoculation of Pan02-luc cells can produce tumor nodules in the pancreas, we inoculated C57BL/6 mice with 10 x 10^6^ Pan02-luc cells. We have evaluated the potential role of the route of cell inoculation by comparing intravenous versus intraperitoneal cell inoculation. The high cell number, 10 x 10^6^ Pan02-luc cells dose was chosen to ensure tumor uptake. The in-live tumor uptake and progression was monitored based on the luciferase activity of Pan02-luc’s bioluminescence intensity (BLI) over 17 days. The data are summarized in Fig 2.

As shown in Fig 2A, only mice inoculated by the intraperitoneal (IP), and not the intravenous (IV), route exhibited tumor uptake notable as BLI signals localized to the upper right side of the body. From day 10 to day 17, IP inoculated mice’s BLI intensity increased significantly (Fig 2A), suggesting a rapid pancreatic tumor growth in mice. Organs from these mice were collected on day 17 and analyzed in situ for Pan02 with BLI marker. The localization of the pancreas BLI level higher than that of other organs is notable and it is consistent with the whole-body BLI mice imaging (Fig 2C). On day 17, we also noticed Pan02 tumor nodules were present in the walls within the peritoneal cavity. Regardless, this observation for IP (and not IV) inoculated mice was consistent with health deterioration reaching the euthanasia criteria.

On day 17, no pancreatic tumor was notable in the IV inoculated group (Fig 2A). Additional studies were done to extend IV inoculated mice observation up to 28 days using identical cell inoculation numbers without significant tumor uptake (S1 Fig). Thus, BLI analysis focused on the IP inoculated group at necropsy as presented in Fig 2B and 2C. We found that tumor nodules were notable and consistent with BLI signals with apparent metastatic spread to multiple organs at necropsy including the peritoneum wall, gastrointestinal tract, omentum, pancreas, liver, spleen, lung and kidney (Fig 2B). Detailed analysis of each collected organ—heart, lung spleen, pancreas, kidney and liver—revealed that the pancreas exhibits the highest intensity of pancreatic tumor consistent with BLI data (Fig 2C). This finding suggests that only IP, but not IV, inoculation of pancreatic ductal carcinoma, Pan02-luc cells in C57BL/6 mice has led to the development of Pan02-induced tumors, predominantly in the pancreas. Therefore, the remaining experiments are done with mice inoculated by IP route.

### The role of inoculated Pan02-luc cell number on pancreatic tumor development

Next, the Pan02 cell-dose effect was evaluated for pancreatic tumor uptake and disease progression. For a pancreatic tumor model intended for therapeutic evaluation, it is critical to identify a minimal and optimal dose that would produce mice with pancreatic tumors that also survive for a sufficient duration to monitor pancreatic cancer disease progression. Thus, we inoculated the mice with 3 cell numbers, 1, 5 or 10 x 10^6^ Pan02-luc cells by IP, and monitored for whole body BLI until the mice reached the euthanasia criteria for termination. The high-dose group inoculated with 10 x 10^6^ Pan02-luc cells provided a means to verify the results presented in the previous section.

**Fig 3:**
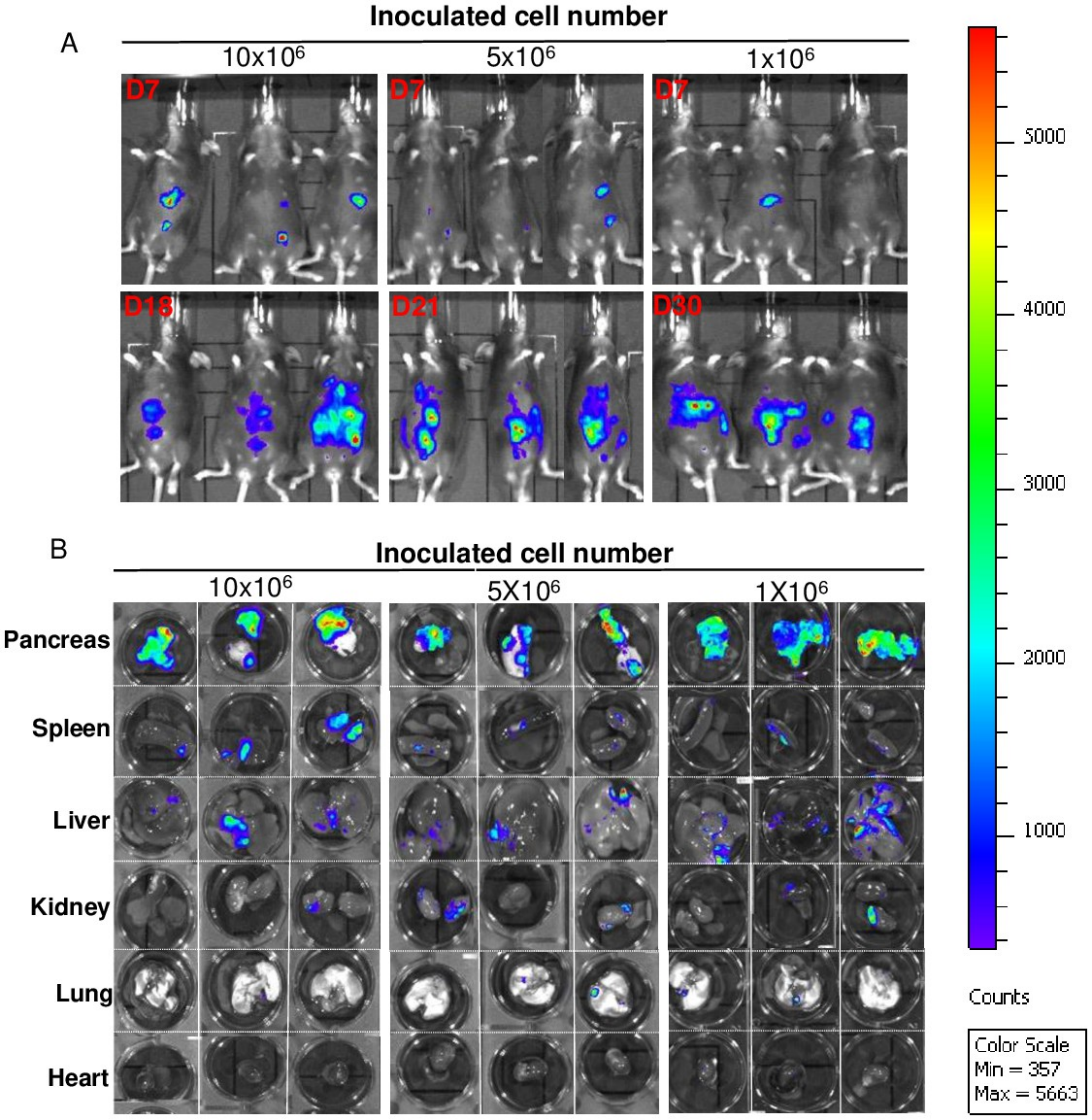
Effects of cell number on pancreatic tumor development. C57BL/6 mice were divided into three groups (n = 3 per group) and intraperitoneally inoculated with different cell numbers of Pan02-luc cells: 1 million, 5 million, or 10 million cells. (A) whole body bioluminescence images of mice were captured on day 7 after inoculation and on the day when mice reached the enthusiasm criteria (day 18, 21, and 30, respectively). (B) Bioluminescence images of pancreas, spleen, liver, kidney, lung, and heart of mice from the three different groups.

By day 7, mice in all 3 test groups exhibited BLI signals, predominantly in the mid-to-upright peritoneal cavity (Fig 3A). The location of the whole-body BLI pattern is consistent with the experiment in Fig 2A. The group of mice inoculated with 10 million cells reached euthanasia criteria at day 18 (Table 2); the group inoculated with 5 million cells reach euthanasia criteria at day 21 (Table 2); and the group inoculated with 1 million cells reach euthanasia criteria at day 30 (Table 2). When mice reached the euthanasia criteria, similar BLI patterns were observed in all three groups (Fig 3A), characterized by intense and spreading BLI localized to mouse abdomens. All mice shared similar clinical symptoms as the pancreatic cancer disease progressed, mainly rapid deterioration starting with precipitate progression in lethargy and inactivity plus rapid decline in health that required euthanasia. However no significant weight change was notable.

**Table 2:** The Pan02 cell inoculation dose and pancreatic tumor disease symptoms requiring mouse euthanasia*.

Pan02 Cell Inoculated	Time to euthanasia
Ix10^7^	18d
5x10^6^	21d
1x10^6^	30d

*C57BL/6 mice were randomly divided into 3 group (n = 3 per group) and intraperitoneally inoculated 10, 5, or 1 million Pan02-luc cells, mice reach euthanasia criteria at day 18, day 21 and day 30 respectively.

At the necropsy of the three groups of mice, we observed the tissue distribution profile of pancreatic tumor detected as BLI positive tissue (Fig 3B) similar to the initial experiment (Fig 2C). As notable in Fig 3B, most of the BLI signal at necropsy is localized in the pancreas of all the mice tested with lower BLI signals found in the liver, spleen and to some extent in the kidney. Minimal or no Pan02 mediated BLI signals were notable in the heart. Very low or no Pan02 BLI was detected in the lung (Fig 3B).

Collectively, these data indicate that while inoculation of Pan02 at 3 dose-concentration ranges may produce pancreatic tumor, the inoculation dose may impact the time-to-tumor development and duration of disease progression. The IP inoculation of Pan02 cells is confirmed to provide good and reproducible tumor development in the pancreas. Comparing the time of three groups of mice that reached the euthanasia criteria, implanting 5 million cells (middle dose) may provide a sufficient time-window (21d) for therapeutic intervention; yet provide early tumor development that can be traced with BLI whole body in-live analysis.

### Characterization of the Pan02 orthotopic pancreatic tumor model

With IP inoculation, Pan02 ductal adenocarcinoma cells can populate tumors in the pancreas of mice. We have further characterized the intraperitoneal Pan02-C57BL/6 in mice as a potential orthotopic pancreatic tumor model. To do so we inoculated 5x10^6^ Pan02-luc (IP) in C57BL/6 mice. We used 5x10^6^ Pan02-luc cells to ensure that all mice developed pancreatic tumors. This approach also enabled testing the hypothesis that intraperitoneally inoculated Pan02-luc initially at home in the pancreas can form pancreatic tumor nodules, and subsequently spread to other tissues/organs. In addition to BLI measurements for semi-quantitative tumor growth tracking, mice were sacrificed on day 7, day 14 and day 21 to collect tissues for histology and to profile changes in serum chemistry. Other clinical signs including weight, lethargy, and general health were also monitored.

### Time-course BLI image and histological analysis suggest initial tumor uptake in the pancreas

#### Pan02-luc pancreatic tumor progression analyzed by bioluminescence intensity (BLI)

**Fig 4:**
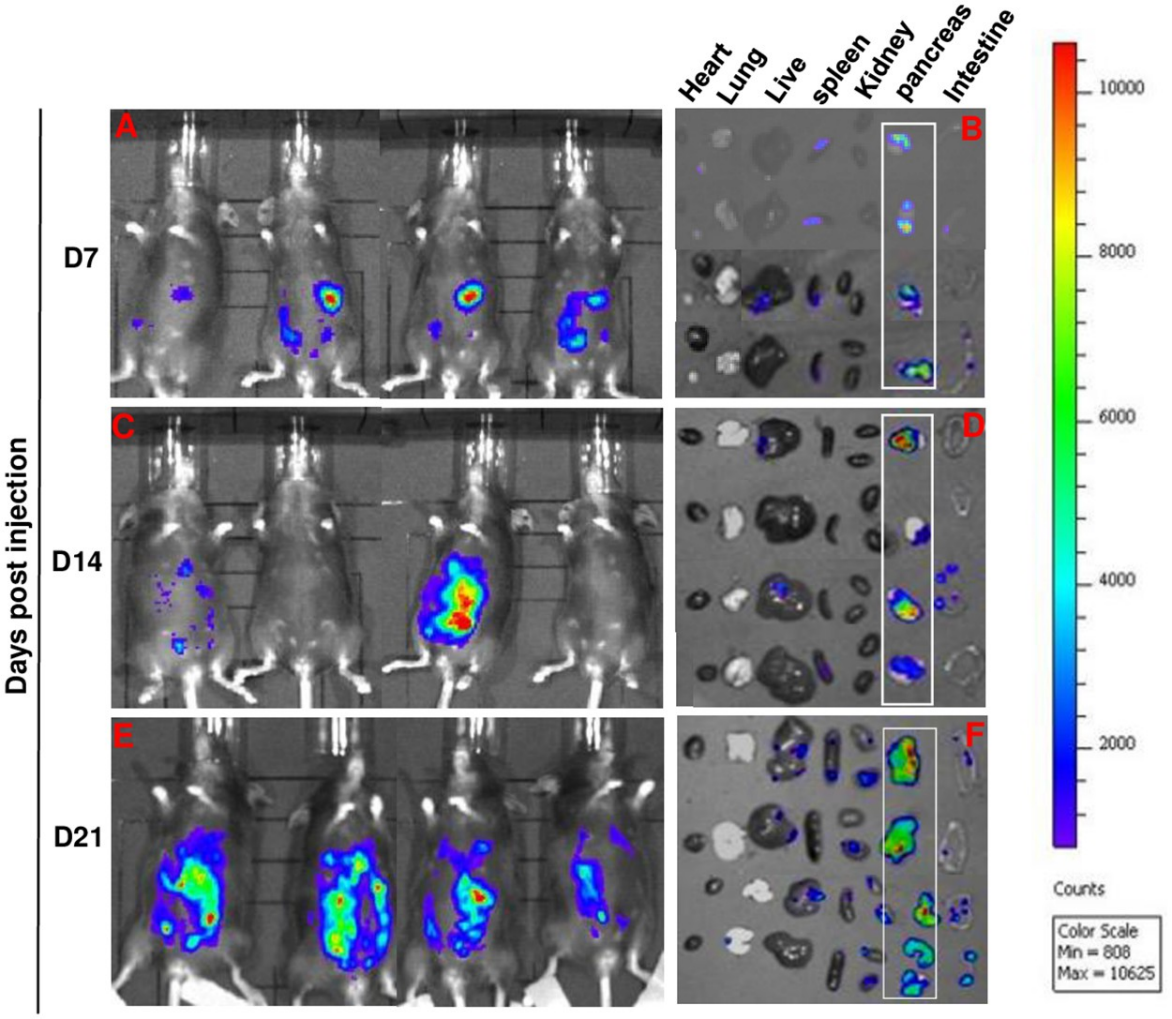
Pan02-luc pancreatic tumor progression analyzed by bioluminescence intensity. C57BL/6 mice (n = 4 per group) were intraperitoneally inoculated with 5 million Pan02-luc cells. (A), (C), (E) Whole body bioluminescence images of mice were captured on day 7, 14, and 21 after inoculation. (B), (D), (F) Bioluminescence images showing the distribution of bioluminescence in the heart, lung, liver, spleen, kidney, pancreas, and intestine of mice on day 7, 14, and 21 after inoculation. The Color scale: Min = 357; Max = 5663.

First, we found that at 5x10^6^ Pan02-luc cells, all mice developed tumors by day 7 based on BLI analysis around the location of the pancreas (Fig 4A) verifying the results of Fig 3 (dose-response study). No BLI signals were detected on lung, heart and kidney (Fig 4B). By day 14 both the whole body and individual organ/tissue analysis indicated other organ/tissue involvement (Fig 4C and 4D). By day 21, additional organs exhibited a lower degree than that of the pancreas suggesting that the Pan02 tumor spread to other secondary target organs and tissues (Fig 4F).

#### Histopathological analysis

**Fig 5:**
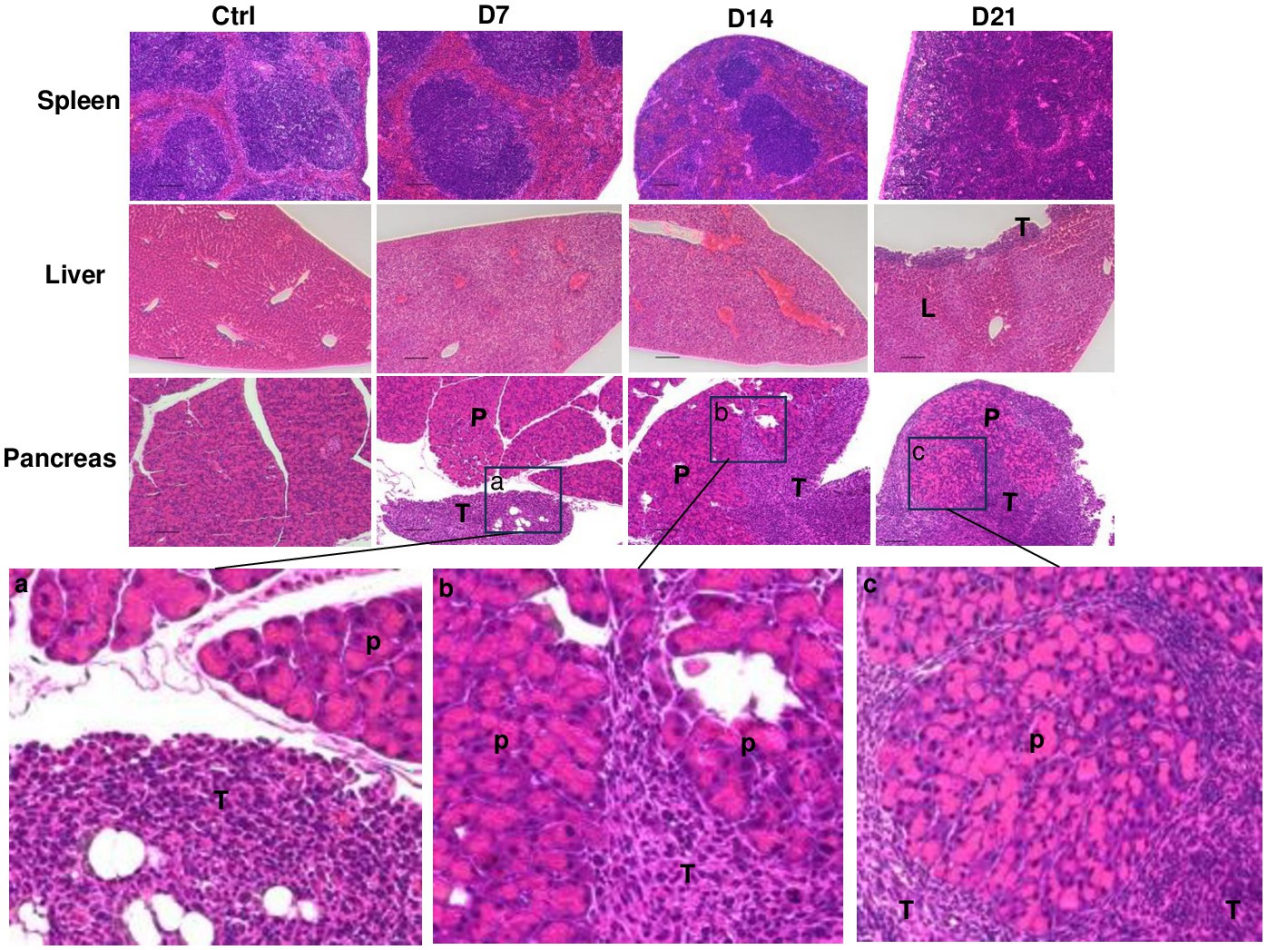
Pathological analysis of Pan02-luc pancreatic tumor progression by Hematoxylin and Eosin (H&E) staining. Representative images of H&E staining in the spleen, liver, and pancreas tissues of the control mice and mice after intraperitoneal inoculation of 5 million Pan02-luc cells for 7, 14, and 21 days. Scale bar: 150 nm. Panel A, B, and C Representative images of H&E staining in pancreas tissues of the mice after intraperitoneal inoculation of 5 million Pan02-luc cells for 7, 14, and 21 days (under 40x lens). “**L**” represents liver tissue, “**P**” represents pancreas tissue, “**T**” represents tumor tissue.

The tissue collected from the Pan02 inoculated C57BL/6 mice on day 7, 14 and 21 were subjected to H&E staining to confirm the presence of the tumor in the mouse tissues. Representative slides for the liver, spleen, and pancreas are presented in Fig 5. For this set of experiments, we have included a control mouse tissue for comparison. The Pan02 cells initially found in the pancreas by day 7 (Fig 5A) exhibited a high density of cells (or hyperplasia) that are distinct from (the larger) pancreatic acinar cells.

Fig 5 also provides the time-progression of Pan02 tumor in the mouse pancreas. On day 7, Pan02 hyperplasia appeared to localize at the periphery of the pancreas (Fig 5A), which progressed to invade pancreatic tissue on day 14 (Fig 5B). By day 21, histology analysis clearly demonstrated that a majority Pan02 hyperplasia appeared to encase pancreatic acinar cells (Fig 5C).

In comparison, the time-based Pan02-related hyperplasia in the spleen and liver appeared to occur later than day 7 with less intensity than that in the pancreas (Fig 5). In the liver, the small tumor nodules were observed adjacent to the surface only on day 21, without invading and destroying the liver normal tissue architecture (Fig 5). The spleen did not show apparent colonization by Pan02-luc cells (Fig 5) until 21 days after inoculation. However, the center spleen’s immune response may have led to drastic change in the spleen structure; notable as progressive loss of red pulp (B cell center), which was virtually overtaken and eclipsed by white pulp by day 21.

**Fig 6:**
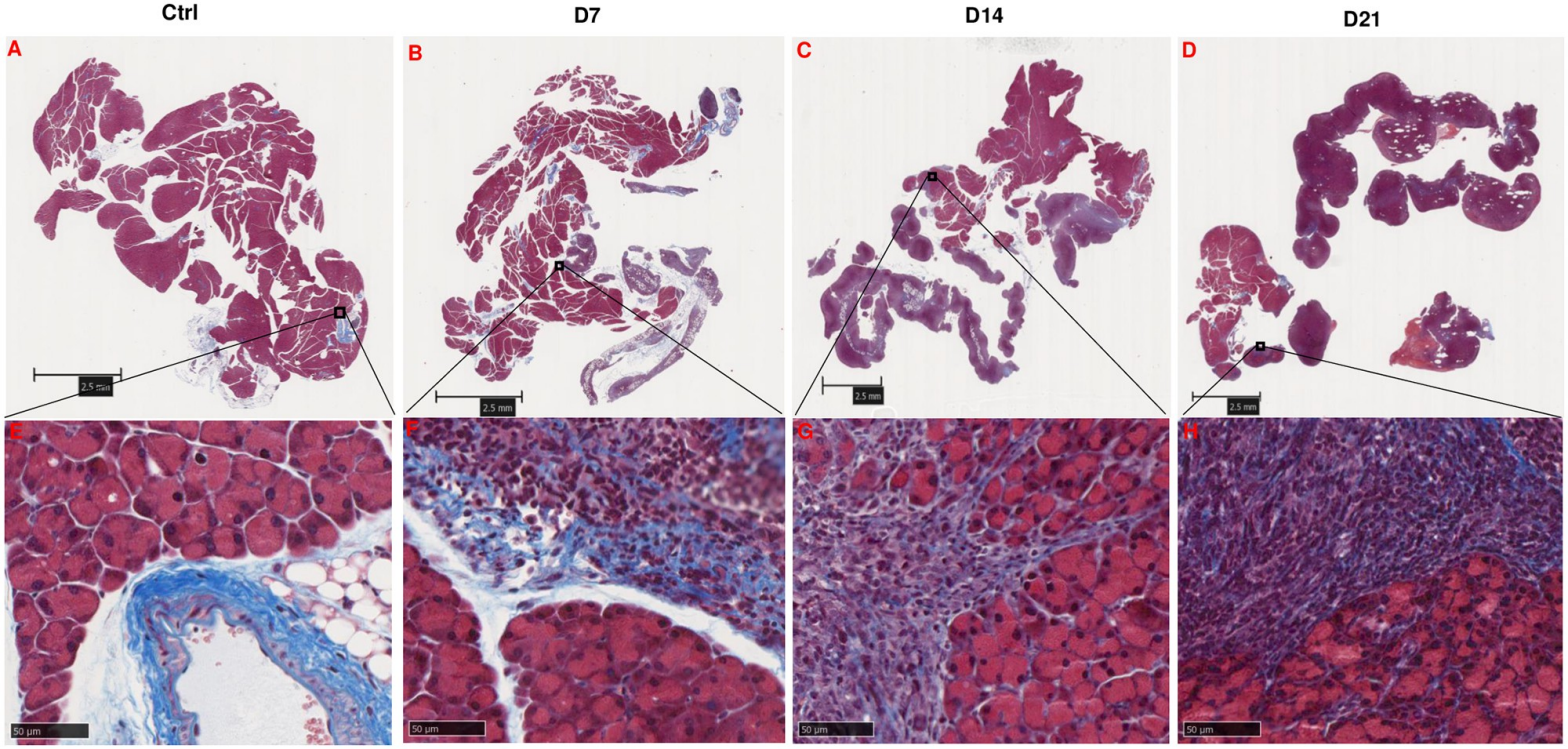
Mice pancreas and Pan02-luc pancreatic tumor Masson’s trichrome staining. Panel A and E: Control mice pancreas Masson’s trichrome staining. Pancreas and Pan02-luc pancreatic tumor Masson’s trichrome staining after IP inoculation 5 million Pan02-luc cells 7 days (B and F), 14 days (C and G) and 21 days (D and H). scale bar: (A-D) 2.5 mm, (E-H) 50 μm.

We next determined tumor tissue desmoplasia, due to extracellular matrix (ECM) contribution to pancreatic tumor mass, in the Pan02 pancreatic tumor. To assess the stromal characteristics of Pan02 tumor nodules, Masson’s trichrome staining [12] was used to detect collagen fibers in the tumor tissues. In the control pancreatic tissue, no significant staining was observed within the pancreatic lobules, with the staining primarily observed in the basement membrane of blood vessels (Fig 6A and 6E). However, in the cancer tissue at day 7, there was a significant deposition of collagen fibers within the tumor stroma, indicating the activation of stromal cells (Fig 6B and 6F). By day 14, noticeable staining was still observed in the tumor stroma, but it appeared that highly proliferated Pan02 cells occupied the space between collagen fibers (Fig 6C and 6G). On day 21, condensed Pan02 cells become more condensed within the nodule, resulting in relatively less ECM staining noted on tumor stroma (Fig 6D and 6H).

Together, these data indicate that (1) intraperitoneally inoculated Pan02-luc initially localized or colonized in the pancreas, and (2) it subsequently spread to other tissues and organs as the tumor metastasized, (3) that Pan02 tumor expanded over time to invade the pancreas and extracellular matrices (ECM) composed of collagens appear to facilitate early but not late stage tumor growth, (4) that tumor mass at day 21 has minimal contribution from (ECM), and (5) that Pan02 tumor growth may elicit changes in the B cell center (red-pulp) in the spleen.

Collectively, these data indicate that a 5x10^6^ Pan02-luc IP inoculation in syngeneic mice produces orthotopic pancreatic tumor consistently by day 7. This orthotopic pancreatic tumor model would be suitable for evaluation of interventional agents. These data also confirm the reproducibility of the model based on IP dosing of Pan02-luc cell homing to the pancreas as a primary tissue target of colonization.

### Serum chemistry of Pan02-luc pancreatic tumor model

To evaluate the impact of pancreatic Pan02-luc tumor growth on serum chemistry, enzymatic, and biochemical changes, we have evaluated several biomarkers that may relate to respective tissues or organs. By day 21, the health condition of the mice deteriorated and reached the euthanasia criteria. The increased blood glucose level aligns with the invasion and destruction of normal pancreas tissue by the inoculated tumor cells (Table 3). Although there was a low degree of tumor cell burden in the liver, there was no significant impact on liver markers, AST, ALT, or cholesterol levels (Table 3). Nevertheless, significant alterations in creatinine, total protein, and sodium levels are noted which may relate to a general decline in health in the mice with pancreatic tumor disease progression (Table 3).

**Table 3:** Serum biochemistry of Pan02-luc tumor-burdened mice at 21 days after inoculation.

Parameters	Biomarker Related to Tissue/Organ	% Change from Day 0	Measured at Day 21	Reference Values (Loeb et al.)
Aspartate Transferase	Liver, muscle tissue	+ 61%	87 U/L	44–221 U/L
Glucose	General, pancreas, stressed tissue	+ 57%	320 mg/dl ↑	60–133 mg/dl
Creatinine	Muscle, kidney	+ 470%	3.4 mg/dl ↑	0.1–1.8 mg/dl
Creatine Kinase	Muscle, general	+ 290%	237 U/L	N/A
Blood Urea Nitrogen	Kidneys, liver	+ 29%	27 mg/dl	2–71 mg/dl
Total Protein	General stressed tissue	- 7.1%	3.9 g/dl ↓	4.6–7.3 g/dl
Alanine Aminotransferase	Liver	0%	27 U/L	14–140 U/L
Sodium	General stressed tissue	- 3.2%	125 mmol/L ↓	153–175 mmol/L
Cholesterol	Liver	+ 15%	69 mg/dl	34–173 mg/dl

↑ represent value increased compared to reference values,

↓ represent value decreased compared to reference values.

## Discussion

While orthotopic mouse models for various cancer types are available for testing therapeutic interventions, such a model for pancreatic tumor uptake, growth and metastatic spread to other tissues has been lagging. Many studies using subcutaneously implanted pancreatic cancer on the skin and those surgically implanted in the pancreas do not provide appropriate physio-pathological context. Thus, the ability to extrapolate results from pharmacokinetic and pharmacodynamic therapeutic effects from the mouse model to estimate therapeutic impact on human pancreatic cancer is limited. Taking advantage of the unanticipated discovery that a simple intraperitoneal, but not intravenous, introduction of pancreatic ductal carcinoma Pan02 cells into syngeneic C56BL/6 mice elicited tumors in native pancreatic tissue (Figs 2–4), we have characterized the time-course tumor progression, clinical sign, and pathological consequences. Results indicate that at 5 x 10^6^ Pan02 cells in inoculant, the pancreatic tumor established in the pancreas by day 7; invading into pancreatic tissues by day 14, and subsequently spread to a majority of the pancreatic organ by day 21. The metastatic spread to other organs at the late stage resulted in a rapid and precipitate decline in health and death. Having such an orthotopic syngeneic model that enables early discernment and late stage disease development could improve the ability to consistently evaluate therapeutic candidates in a physiologically relevant pancreatic tumor development and progression model. Leveraging on the luciferase marker expressed in Pan02-luc cells, we have characterized in detail the time-course and dynamics of this orthotopic pancreatic ductal carcinoma that induces pancreatic tumors in mice. The Pan02 cells introduced in the peritoneal cavity are first home primarily to the pancreas; subsequently grow, invade and overtake the pancreas, before spreading to liver, kidney and to some extent lungs at later stages (Fig 5). The formation of peritoneal metastases at late stage of the disease was noted.

The value of having a syngeneic orthotopic mouse model that exhibits 100% tumor uptake in a reproducible manner cannot be underestimated. We found that with a 5x10^6^ Pan02 inoculant all mice exhibit tumor nodules in the pancreas and consistently exhibit clinical progression in about 3 weeks. The homing of Pan02 to the pancreas and pancreatic tumor development and progression is verified to recapitulate the clinical changes, histological morphology, and the stromal tumor microenvironment of pancreatic ductal adenocarcinoma, in a reproducible manner. In addition, as a syngeneic model that does not involve surgery-related inflammatory responses, this orthotopic pancreatic tumor model may better mimic human pancreatic tumor progression. Having such a model may enable evaluation of new therapeutic strategies in an accelerated manner with potentially enhanced confidence in predicting clinical disease progression. With these unique characteristics, drug and other immunologic interventions can be tested for early or late stage effects on pancreatic cancer development and progression according to their respective oncogenic and immunologic interactions.

Previous studies with Pan02 cells have used intraperitoneal routes of inoculation [13–19]. However, these studies were intended to evaluate tumor development in the peritoneal cavity and have not systematically evaluated tumor establishment in the host tissue, the pancreas. For example, Greco et al. [19] measure Pan02 tumor distribution mostly in the visceral organs and peritoneal cavity only after three weeks post inoculation. This data is consistent with our finding that, by 3 weeks peritoneal Pan02 metastasis are noted in the liver. Unfortunately Greco et al. intent was to evaluate TGF-beta at a later stage of disease (cachexia) or wasting syndrome. Thus no information on whether the pancreas is even involved in the reported data. With time-course systematic tracking of tumor kinetics and tissue localization studies (Figs 4–6), we were able to systematically elucidate pancreatic uptake, tumor growth and tumor progression during early stage of orthotopic Pan02 tumor over the first seven-days, offering a comprehensive account of its initial stages, which may be critical for developing therapeutic strategies for early interventions.

Secondly, while Greco et al. established a syngenetic pancreatic cancer cachexia model and demonstrated tumor metastasis to the liver and mesenteric implants, they did not conduct an examination of tumor presence within the pancreas itself. In contrast, in our current study, we constructed an orthotopic pancreatic cancer model, predicated on the observation that the inoculated Pan02 cells initially established in the pancreas in a consistent pattern. Consequently, these reports focus on evaluation of the peritoneal tumor nodule and the histology and metastasis of pancreatic tumors, which are formed around 2 or 3 weeks after inoculation. The systematic studies with Pan02-luc, in-live time-course event tracking of tumor development, growth and spread in this report provide a means to verify (1) the initial homing site of Pan02 cells, (2) tumor uptake, growth and spread to other visceral organs, and (3) comprehensive characterization of the histology and stromal characteristics of early-to-late-stage pancreatic tumor in this orthotopic pancreatic tumor model. This study, to our knowledge, is the first to produce an orthotopic pancreatic tumor model in syngeneic mice by simple inoculation of a pancreatic adenocarcinoma cell to produce pancreatic tumor in all mice in the pancreas that recapitulate many of the clinical symptoms and pathology of pancreatic cancer in humans. These remarkable characteristics of this orthotopic pancreatic tumor model are notable.

Even though Pan02-luc cells expressing the luciferase marker that enable in-live or real-time tracking of pancreatic tumor development and progression, they may exhibit differential drug sensitivity of the cancer cells. In a limited study using a current pancreatic cancer drug, gemcitabine that interferes with DNA synthesis, we found that Pan02-luc exhibits similar sensitivity to that in the parent Pan02 cells (Table 1). Additional studies with other chemotherapeutic agents are of interest. However, such studies including drug combination effects are beyond the scope of this report.

Subcutaneously implanted pancreatic tumor models [20] and genetically engineered pancreatic tumor mouse models (GEMMs) [21] are available as non-surgical models for therapeutic evaluation. While widely used for therapeutic evaluation, including easy access to intra-tumoral drug injections, subcutaneous pancreatic tumor did not reflect pathophysiologic microenvironments within the pancreas, which may relate to replicating the metastatic potential observed in humans [20]. With specific sets of gene knockout or over-expression in mice GEMMs providing specific gene modulation related to tumor developed and progression, it may elucidate specific oncogenic pathways. However, the use of GEMMs as a preclinical model for therapeutic evaluation may be limited by (1) the fraction of mice that develop pancreatic tumor, (2) asynchronous tumor growth rates for discerning placebo vs treatment outcomes, and (3) restrictions to a narrow set of specific variations in genes (and products). In addition, pancreatic tumor models, based on GEMMs typically exhibit limited metastasis, which restricts their utility in studying the metastatic aspects of pancreatic cancer and evaluating therapeutic interventions targeting metastatic disease. While the genetics the Pan02 cells remain to be fully elucidated, Pan02 (also known as Panc02) cells are reported to carry many common mutations in human pancreatic cancers, including Muc1 and Muc4 [22]. Genes coding mucin proteins that can be overexpressed lead to the commonly seen desmoplasia, or overgrown extracellular matrix proteins in human patients. The mutations of KRAS and SMAD4, which are key genes identified in human pancreatic cancer, have also been detected on Pan02 cells [23]. In the present study, the intraperitoneal inoculation of Pan02 cells demonstrates distinct advantages over other models. Specifically, the Pan02 cells exhibited a unique ability to home in the pancreas, leading to the formation of tumor nodules at an early stage. This aspect closely resembles the clinical scenario where pancreatic tumors develop and progress within the pancreas before spreading to other sites. The observed proliferation, invasiveness, and metastatic potential of the Pan02 cells recapitulates the characteristics of pancreatic tumor development and also rapid progression to advanced-stages in patients with pancreatic cancer. In addition to the pathological evidence, the similarity between the rapid decline in health at the end stage (Table 2) and the late-stage symptoms observed in patients provides a means to capture end-stage clinical disease progression with this model.

It is not clear why the introduction of Pan02 cells by the peritoneal, and not the intravenous, route led to consistent tumor establishment and in mice. It is likely that Pan02 cells introduced into the blood after intravenous dosing may not be readily accessible through the tight junction of endothelial cells lining the blood vessels to reach the pancreatic cells and tissues. This step may be necessary to support the pancreatic ductal adenocarcinoma, Pan02 cells. In contrast, as a visceral organ in the peritoneal cavity, the Pan02 cells may have gained access to pancreas cells and tissues via more porous epithelial cell lining encasing the pancreas facing the peritoneal cavity that enable Pan02 to gain access to the pancreas [24]. Whether other pancreatic ductal carcinoma (such as PC1, PDPPaCa, HT2, HPC) [25] can be substituted for Pan02 to develop a pancreatic tumor model is not known and it is of our current interest. While the exact mechanism of Pan02 homing to the pancreatic tissue and invasion of the pancreas until eventually filling nearly the organ with hyperplasia remains elusive, it is clear that the pancreas is the first organ or tumor attachment and that it supports growth and progression. Given the evolving knowledge of markers and determinants that enable pancreatic cells to dock and establish in the tissue origin of Pan02, it may provide a tool to explore biomarkers or key molecules for serving as a druggable target for pancreatic cancer. Additional mechanistic studies, while interesting, are beyond the scope of this report.

It is interesting to note that at the time of diagnosis, >80% of the patient with confirmed pancreatic cancer may have already advanced to the metastatic disease stage. Thus, the inability of early diagnosis with appropriate marker(s) and disease symptoms may have contributed to the high morbidity and mortality of pancreatic cancer [26]. Current Pan02 orthotopic pancreatic tumor models can be used to discern both earlier delineation and late stage analysis of pancreatic tumor development over the 21 days. This may allow molecular and cellular dynamics in the pancreas related to the formation of tumor nodules within the pancreas—before spreading metastatically to liver, kidney and lung within tissues of peritoneal cavity—to be analyzed. It may also be of interest to probe why Pan02 cells were not able to establish tumor nodules in the blood accessible organs such as the lung and spleen when given by intravenous route (at high Pan02 cell inoculation). Only when given through a peritoneal route did Pan02 cells gain access to the pancreas, perhaps through the more porous epithelial cells lining the pancreas within the peritoneal cavity. This observation may also be relevant in consideration of the route of delivery for therapeutic agents to gain access to the pancreatic tissues in their first passage. Intravenously administered drugs may be subjected to similar access limiting with that observed for Pan02 cells in our studies.

In summary, we have developed and characterized a novel and high efficiency orthotopic pancreatic tumor model based on a one-step Pan02 pancreatic ductal carcinoma inoculation into the peritoneum. As immortalized Pan02 cell lines are derived originally from C56BL/6 mice, this syngeneic orthotopic pancreatic tumor model may be suitable for evaluating intervention based on both drug and immunologic modalities. The consistent and predictable tumor uptake, growth and invasion in the pancreatic tissues before spreading to other visceral organs may also serve as a tool for the dynamics of host and pancreatic cancer cells to support research in pancreatic cancer biology. Regardless, this orthotopic Pan02-luc pancreatic tumor model may serve as a simplified and physiologically relevant pancreatic tumor model to evaluate promising therapeutic interventions in an accelerated and immune competent manner.

## Supporting information

S1 Fig Tumor establishment at 28 days after intravenous inoculation Pan02 cells. C57BL/6 mice (n = 4) received an intravenous (tail vein) inoculation of 5 million Pan02- luc cells. Whole body Bioluminescence images of mice were captured on day 28 after inoculation using the IVIS Spectrum System. Images were taken 15 minutes after intraperitoneal injection of D-luciferin, with a 3-minute exposure. The Color scale: 241 Min = 88; Max = 10625.
(TIFF)
