# Supplementary figures and images for: Inoculation of Pan02 cells produces tumor nodules in mouse pancreas: Characterization of a novel orthotopic pancreatic ductal adenocarcinoma tumor model for interventional studies
Source: PLoS One. 2024 Mar 28;19(3):e0300723. doi: 10.1371/journal.pone.0300723 (PMC10977750; doi:10.1371/journal.pone.0300723)

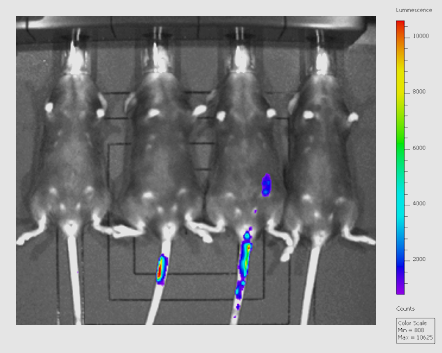

Supplement: S1 Fig — C57BL/6 mice (n = 4) received an intravenous (tail vein) inoculation of 5 million Pan02- luc cells. Whole body Bioluminescence images of mice were captured on day 28 after inoculation using the IVIS Spectrum System. Images were taken 15 minutes after intraperitoneal injection of D-luciferin, with a 3-minute exposure. The Color scale: 241 Min = 88; Max = 10625. (TIFF) [file pone.0300723.s001.tiff]
